# Investigation of factors affecting the sound absorption behaviour of 3D printed hexagonal prism lattice polyamide structures

**DOI:** 10.1038/s41598-024-81496-7

**Published:** 2024-12-28

**Authors:** Martin Vašina, Jakub Měsíček, Martin Mánek, Quoc-Phu Ma, Jiří Hajnyš, Jana Petrů

**Affiliations:** 1 Faculty of Mechanical Engineering, Department of Machining, Assembly and Engineering Metrology, VSB-Technical University of Ostrava, Ostrava-Poruba, 708 00 Czech Republic; 2 Faculty of Technology, Department of Physics and Materials Engineering, Tomas Bata University in Zlín, Zlín, 760 01 Czech Republic; 3 Faculty of Mechanical Engineering, Department of Applied Mechanics, VSB-Technical University of Ostrava, Ostrava-Poruba, 708 00 Czech Republic

**Keywords:** Sound absorption, 3D printing, Selective laser sintering, Lattice structure, Specific airflow resistance, Ansys software, Environmental sciences, Engineering, Materials science

## Abstract

The aim of this work is to investigate the sound absorption properties of open-porous polyamide 12 (PA12) structures produced using Selective Laser Sintering (SLS) technology. The examined 3D-printed samples, fabricated with hexagonal prism lattice structures, featured varying thicknesses, cell sizes, and orientations. Additionally, some samples were produced with an outer shell to evaluate its impact on sound absorption. Experiments were conducted using the transfer function method with an acoustic impedance tube in the frequency range of 250 Hz and 6400 Hz. The results showed that the studied geometric factors significantly affected the sound absorption of the PA12 samples. In some cases, the hexagonal prism lattice structures demonstrated relatively high sound absorption properties. Thanks to their properties such as lower weight, recyclability, and resistance to moisture and chemicals, these structures become competitive with commonly used sound-insulating materials, making them promising candidates for sound absorption. Furthermore, numerical simulations using Ansys software confirmed that the sound absorption properties of the open-porous material structures generally increased with higher specific airflow resistance. The findings highlight the advantages of 3D printing technology in producing complex, highly customizable porous structures for noise reduction applications.

## Introduction

Noise is usually defined as an unwanted sound or combination of sounds that can have an undesirable effect on people. It can cause physiologic damage or psychological harm and may manifest in the form of physiological stress reactions, adverse social consequences, sleep disturbances and detrimental economic impacts^1^. Therefore, it is necessary to reduce noise pollution by appropriate measures. In general, noise control measures include active control and passive control^2^. The active noise control (ANC) system is an electro-acoustic device that is based on the principle of destructive interference by generating an anti-noise of the same amplitude and opposite phase compared to the unwanted noise. The ANC system can eliminate noise at low frequencies^3^. On the contrary, passive noise control (PNC) systems, which use sound-absorbing materials, are effective for reducing mid- and high-frequency noise^4,5^.

Sound-absorbing materials are widely used to reduce noise in various areas. The principle of noise reduction is the conversion of acoustic energy into heat using suitable sound-absorbing materials^6^. In general, materials with porous, spongy, and fibrous structures are characterized by good sound absorption properties^7–9^. There are two main types of sound absorbers, namely resonant and porous sound-absorbing materials^10,11^. Resonant sound absorption materials, including Helmholtz resonators, membrane absorbers, and perforated panels, operate on the principle of the internal resonance effect. However, these materials usually exhibit good sound absorption properties only in a narrow frequency range at low frequencies. Porous sound absorption materials consist of channels, cracks, and cavities that allow acoustic waves to enter the materials. The incident acoustic energy is then dissipated into heat, generated by the friction of air molecules against the pore walls, and viscous losses incurred by the airflow’s viscosity within the materials. Compared to resonant absorption materials, porous materials can absorb sound across a broader frequency range^12^. Furthermore, porous sound absorbers are characterized by extraordinary properties such as low price, easy molding, and weight reduction^10^. The ability of a material to absorb sound is influenced by a variety of factors^13–16^ including excitation frequency, material thickness, density, porosity, airflow resistivity, tortuosity, perforation, the depth of the back cavity, combination of materials, the angle of the incident acoustic wave, surface shape, and temperature, among others.

Many researchers have also investigated the sound absorption ability of porous materials made using 3D printing technology. Carbajo et al.^17^investigated the sound absorption properties of 3D printed macro-perforated PLA (Polylactic Acid) specimens. Their findings revealed that the macro-perforated samples showed enhanced sound absorption compared to non-perforated PLA samples. This improvement was attributed not only to quarter-wavelength resonance but also to the effects of diffusion phenomena and the influence of double porosity. Better sound absorption can be achieved by using 3D printed polymeric PLA multilayered polymer microchannels^18^. In this case, the 30 mm-thick multilayered microchannels exhibited subwavelength behavior with near perfect broadband absorption in the target frequency ranges (with an absorption average up to 0.87). Different configurations of 3D-printed perforated panels combined with polyurethane foam^19^were studied by Patil et al. Suguhara^20^ investigated the sound absorption of 3D printed porous structures composed of numerous horizontally and vertically interconnected resonators, made from a dedicated ceramic blended resin. Furthermore, a grid was attached to the resonator holes, resulting in a high sound absorption coefficient across a wide area, similar to that of widely used fiber-based porous sound-absorbing materials, such as glass wool and rock wool. Therefore, sound-absorbing materials consisting of micro-periodic structures based on resonator-type unit cells can be used in practice as a substitute for commonly used fiber-based sound insulation materials. However, the sound absorption efficiency of these materials decreases due to humidity, weather conditions and gravity. Liu et al.^21^ demonstrated the impact of perforation angles on the sound absorption of additively manufactured porous polycarbonate materials. The findings revealed that as the perforation angle increased, there was a gradual decrease in both the peak sound absorption coefficient and the corresponding frequency of the polycarbonate samples.

The cell size also significantly influences the sound absorption properties of porous materials. Zieliński et al.^22^ indicated that an increase in cell size led to a decrease in the sound absorption of open-cell foams. The sound absorption properties of 3D-printed bio-degradable panels manufactured from three types of PLA materials with three internal profiles (circular, triangular, and corrugated) and at five filling densities were studied by Zaharia et al.^23^ It was found that the triangular profile showed the best sound damping performance for the three types of studied materials. Monkova et al.^24^ conducted a study on 3D printed open porous ABS materials and investigated different structural types, including Cartesian, Octagonal, Rhomboid, and Starlit. Their findings showed that ABS acoustic absorbers made with the Starlit structure exhibited a higher ability to damp noise compared to other ABS structures examined. Furthermore, the sound absorption properties of the ABS samples improved with an increase in both the sample volume ratio and material thickness. Additionally, enlarging the air gap size behind the porous ABS samples enhanced their sound absorption properties, particularly at lower excitation frequencies. This phenomenon was also observed in the sound absorption study of multilayer sound absorbers^25,26^ including a micro-perforated panel absorber (MPPA) layer, a porous material layer and an air gap, as well as in the case of 3D-printed polymer multilayer micro-perforated panels containing multiple air gaps^27^. Rezaieyan et al.^28^ compared the sound absorption performance of natural fiber reinforced composite micro-perforated panels (NFRC-MPP) made from cork fiber and polylactic acid (PLA) with conventional PLA-MPP panels manufactured using 3D printing. The results showed that the average sound absorption coefficient (SACA) of the NFRC-MPP sound absorbers was 25% higher compared to conventional MPP sound absorbers. A significant increase in the SACA was achieved by adding a layer of kenaf porous material behind the MPP and simultaneously introducing a cavity between the two layers and a cavity behind the kenaf absorber. Arjunan et al.^29^ studied the acoustic properties of titanium (Ti6Al4V) micro-perforated panels (MPPs) in maze, hexagonal, and star designs. It can be concluded that these metallic MPPs generally exhibited low sound attenuation properties. However, the ability to damp noise was significantly increased for MPPs backed with 25 mm thick polymeric foam at the frequency *f* > 600 Hz. In both cases, the best sound absorption was achieved with the MPPs in the star design followed by the maze and hexagonal designs. Goh et al.^30^ investigated the sound-absorbing properties of 3D printed sandwich core panels, which consisted of a fiberglass face sheet and a core. Specifically, they tested three core designs: hybrid honeycomb, double ellipse, and corrugated triangle with horizontal beam cores. It was found that the sandwich structures generally exhibited lower acoustic absorption performance compared to the respective core structures. Cavalieri et al.^31^ studied the sound absorption performance of a porous 3D printed PLA material where multiple split-ring resonators or circular non-resonant inclusions were embedded in the transversely isotropic porous layer. The findings indicated that the porous material itself exhibited lower sound absorption properties compared to transversely embedded resonators and inclusions. Li et al.^32^ optimized the structural composition of heterogeneous porous auxetic absorbers to achieve an average broadband sound absorption coefficient of 0.77. Relatively high sound absorption peaks (*α*_*max*_≅ 0.85) were found for extremely tortuous 3D-printed sound absorbers with labyrinthine channels in solid skeletons^33^. However, the high sound absorption was not broadband but rather localized in a narrow frequency range around the peaks. On the contrary, excellent sound absorption performance was obtained in a 3D-printed thin-walled mesoscopic hybrid slit-resonator metamaterial absorber^34^. Zieliński et al.^35^ explored how different 3D printing technologies affect the sound damping properties of porous samples. The study revealed that samples fabricated using Color Jet Printing exhibited superior noise damping capabilities. Conversely, samples produced via Stereolithography technology demonstrated the least effective sound absorption properties.

Currently, the development of mathematical simulations is a popular trend across various research areas, including the study of frequency dependencies of the normal incidence sound absorption coefficient. There is a wide range of theoretical models for simulating the sound absorption of porous sound-absorbing materials. The theoretical models are classified into empirical and phenomenological models^7,13^. Empirical models require only air flow resistivity, whereas phenomenological models require additional parameters such as porosity, viscous characteristic length, thermal characteristic length, and tortuosity of a given porous material. The empirical models with a macroscopic view are considered as a simple model for fast approximation using power-law relations through best fitting of a large quantity of experimental data. There are many theoretical models to predict the acoustic behavior of sound-absorbing materials, including the Delany-Bazley, Voronina, Berardi, Ramis, Biot-Allard, Johnson-Champoux-Allard, and Miki models^7,13,36^. However, various limiting factors affect their applications, including specific types of materials (such as multilayer porous structures), frequency range, porosity, pore shape and size, and fiber diameter. Consequently, these factors significantly impact the accuracy of mathematically simulated results obtained using the given theoretical model compared to experimental measurements.

The aim of this paper is to experimentally investigate various factors affecting the sound absorption properties of polyamide structures with hexagonal prismatic lattice structures fabricated using the SLS technology. The material studied is PA 2200, also known as PA12, whose mechanical properties and fabrication processes are well-documented in the existing literature, demonstrating its suitability for our applications^37–39^. In addition to the factors that are already mentioned (i.e., sample thickness, excitation frequency, cell size, and air gap), the influence of the outer shell and the orientation of hexagonal lattice cells on the sound insulation properties was investigated. This paper highlights the flexibility offered by 3D printing technology in customizing design features and producing complex structures, such as lattices, while also allowing for adjustments in their orientation to facilitate timely experimental verification. To the best of our knowledge, no relevant studies investigating the sound absorption properties of the above types of 3D printed materials with respect to these factors have yet been published. Finally, the sound absorption properties of the investigated hexagonal prismatic lattice structures were consistent with the airflow resistance results obtained from numerical simulations using Ansys software.

## Materials and methods

### Production of 3D printed samples

In this study, EOS PA 2200 (also known as PA12), a thermoplastic material developed by EOS GmbH (Krailling, Germany), was used as the primary material for fabricating 3D-printed samples using SLS technology. This material is widely used in the industry, and its production and post-processing are the subject of extensive research. The mechanical properties of this thermoplastic material, including its melting point, which represents the temperature at which the solid and liquid phases are in equilibrium^40^, are given in Table 1^41^.

**Table 1:** Basic properties of PA12 material.

Density of laser sintered parts [kg∙m^−3^]	Young´s modulus of elasticity [MPa]	Tensile strength [MPa]	Elongation at break [%]	Melting point [°C]
930 ^*^	1650 ^*^	48 ^*^	18 ^*^	176 ^*^

^*^ According to manufacturer´s (EOS GmbH, Krailling, Germany) data sheets.

The EOS FORMIGA P 110 Velocis 3D printer (EOS GmbH, Krailling, Germany), equipped with a CO₂ laser with a maximum rated power of 30 W, was used to produce the tested samples. The machine dimensions were 200 mm × 250 mm × 330 mm, with a scanning speed of up to 5 m·s⁻¹. EOS PA 2200 powder was available for producing functional prototypes using L-PBF technology. The powder was a 50:50 mixture of new and previously used material of the same type. This mixing ratio, known as the refresh factor, was recommended by the powder manufacturer. The 3D printing parameters are listed in Table 2.

**Table 2:** 3D printing parameters.

Layer Thickness	Process Chamber Temperature	Removal Chamber Temperature	Beam Offset	Material Dependent Scaling			
				X	Y	Z (0 mm)	Z (300 mm)
[µm]	[°C]	[°C]	[mm]	[%]	[%]	[%]	[%]
100	168	150	0.28	2.96	2.98	2.60	2.00

**Fig. 1:**
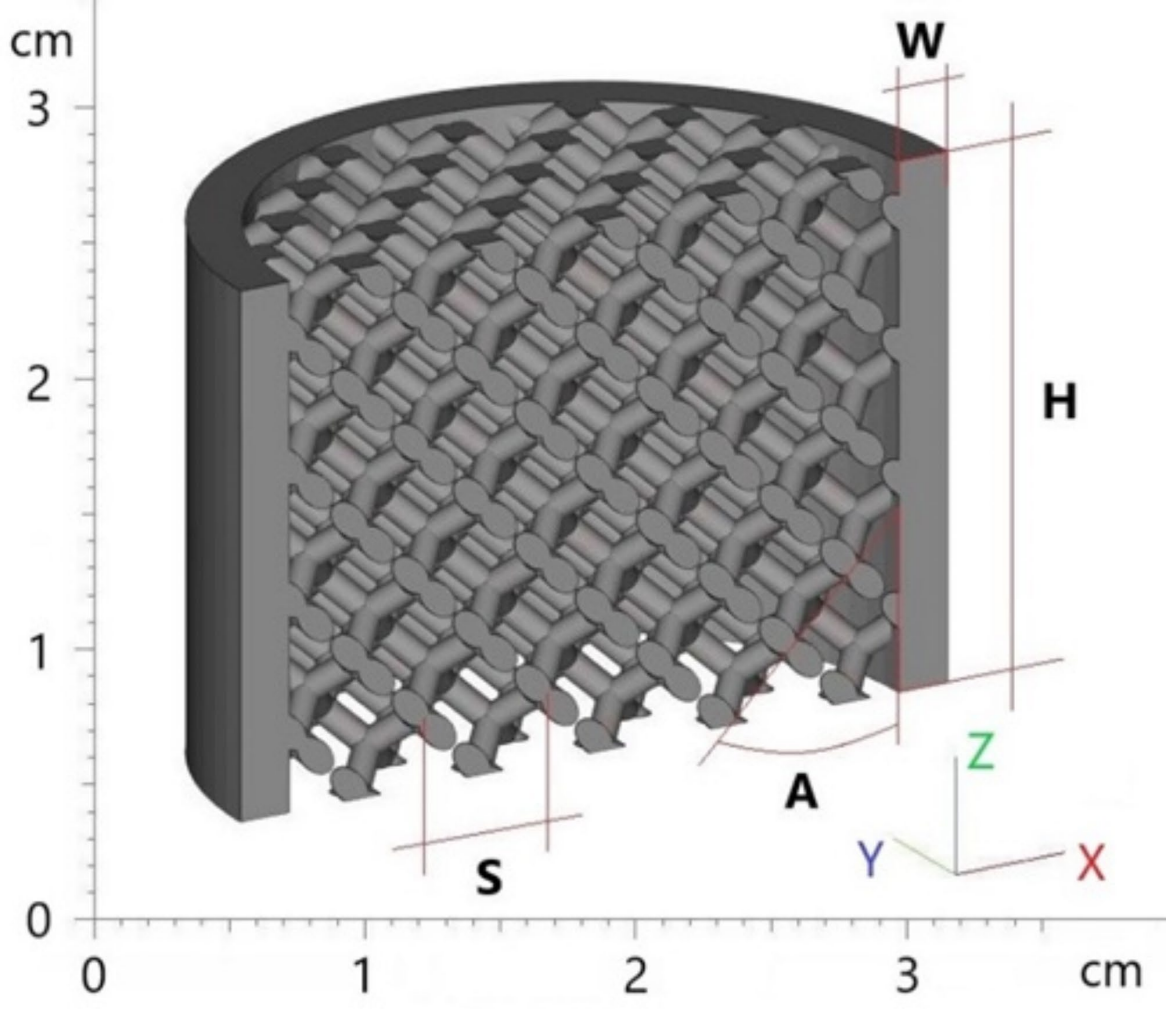
Design parameters (i.e., *A*, *H*, *S*, *W*) of 3D printed circular specimens.

3D printed samples were designed as circular lattice structures of the “Diamond 20% Relative Density (msg)” type, using Materialise Magics software. The samples, a schematic section of which is shown in Fig. 1, were made with an outer diameter of 28.9 mm and five different heights (*H*), namely 10 mm, 20 mm, 30 mm, 50 mm, and 80 mm. To analyze the effect of cell size (*S*) on the sound absorption properties, samples of five different cell sizes (i.e., 5 mm, 7 mm, 10 mm, 13 mm, and 15 mm) were created. The selection of the heights and cell sizes mentioned above was based on the need to investigate a range of geometries that could significantly impact the sound absorption properties of the studied hexagonal prism lattice structures. These specific values were chosen to include both small and large variations in height and cell size, allowing for a comprehensive analysis of their effects on acoustic performance. Additionally, these dimensions are practical for 3D printing and relevant to potential real-world applications, where different structural thicknesses and cell sizes can influence overall efficiency and material usage in sound absorption technologies. The selection aimed to balance experimental feasibility with the need to explore a broad spectrum of geometric configurations.

The samples were also fabricated with four different angles (*A*) of cell orientation in the material structure, namely 0°, 15°, 30°, and 45°. In addition, the samples under investigation were manufactured both without and with a circular wall (*W*) of 2 mm thickness. Thus, a total of 200 pieces of 3D printed samples were produced. After the 3D printing process, the fabricated samples were inspected to verify their geometric dimensions.

**Fig. 2:**
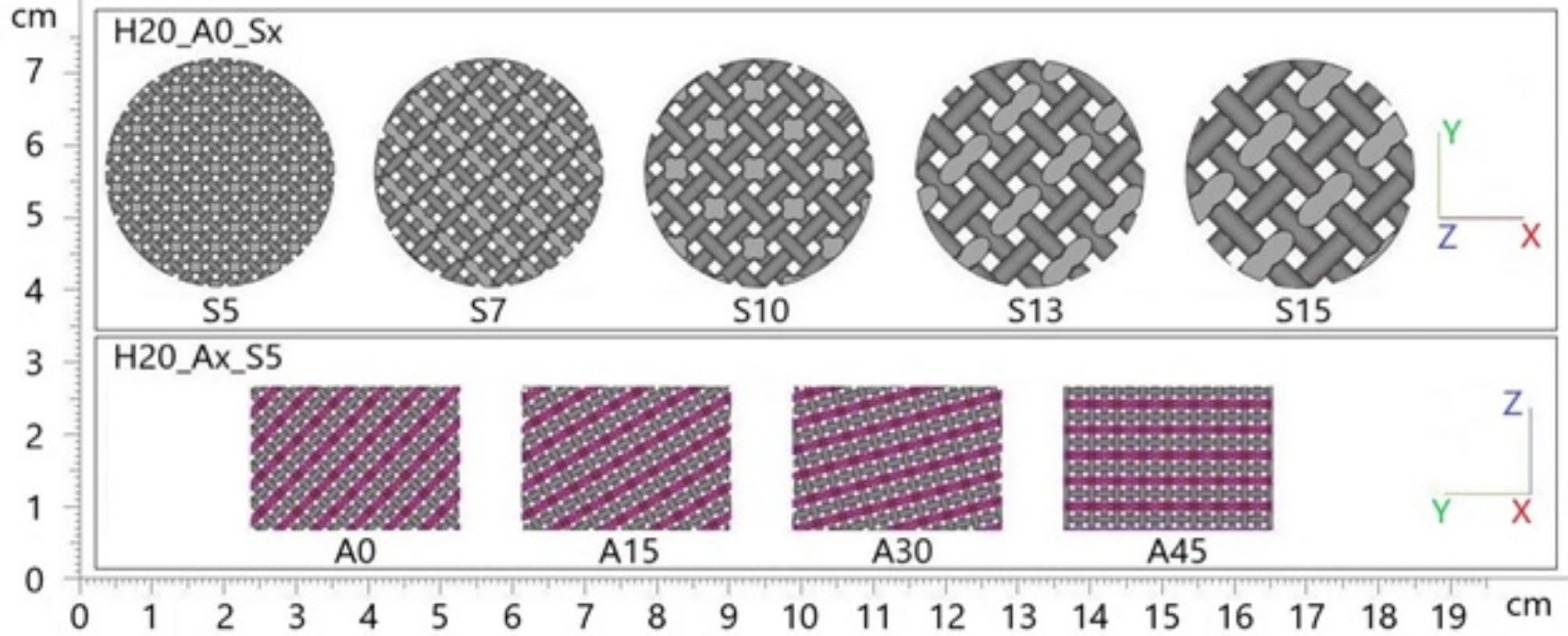
Geometric variants with different design parameters.

In the sample designations, the length dimensions (i.e., *H*, *S*, *G*, and *W*) are given in millimeters (mm), and the angle *A* is given in degrees. The symbol “*x*” in the sample designation denotes the variable being evaluated.

**Fig. 3:**
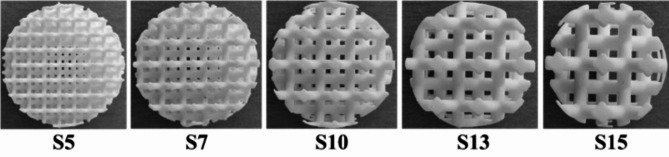
Photo of 3D printed tested samples of type H30_A0_Sx with various cell sizes *S* (in mm).

**Fig. 4:**
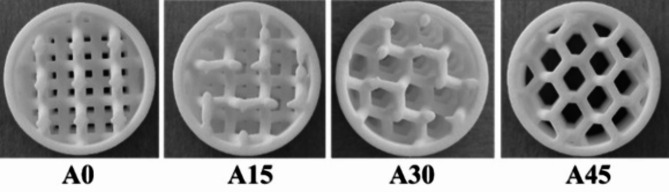
Photo of 3D printed tested samples of type H30_Ax_S10_W2 with various rotation angles *A* (in °) of the hexagonal lattice cells.

**Fig. 5:**
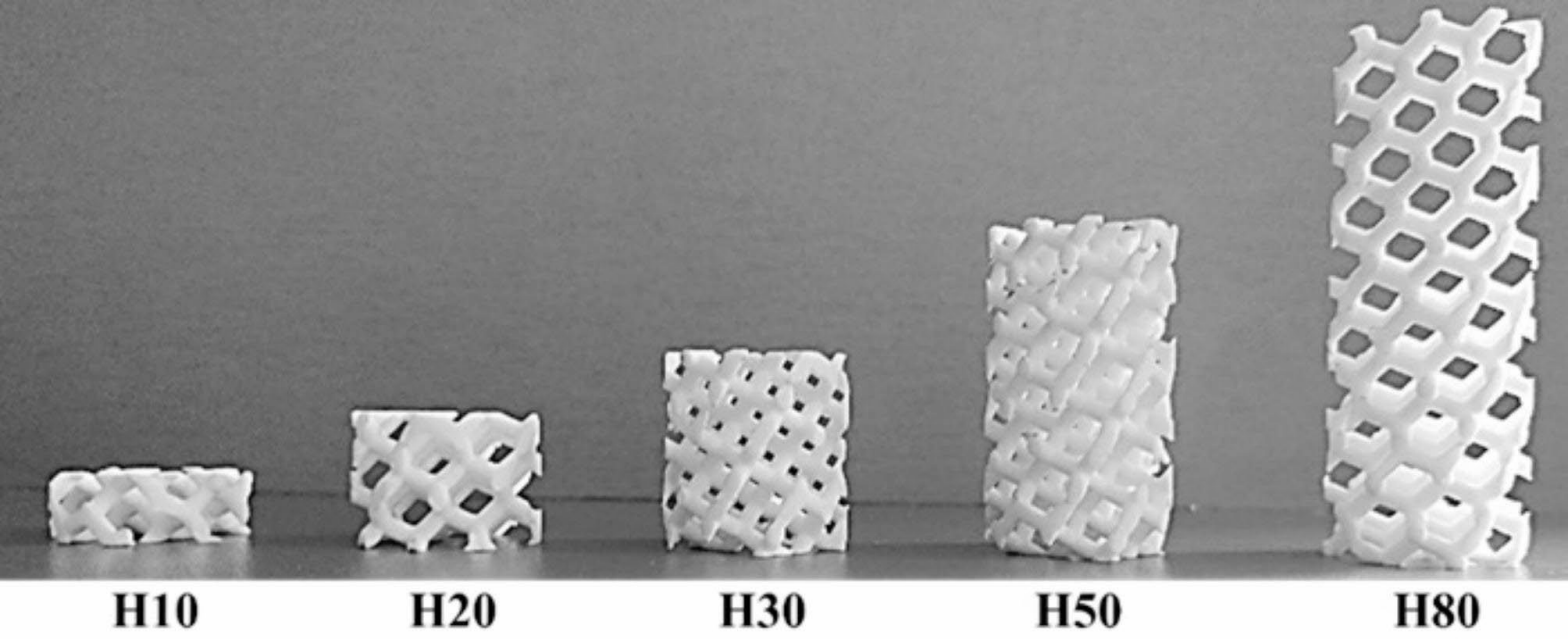
Photo of 3D printed tested samples of type Hx_A15_S13 with various heights *H* (in mm).

The effect of the cell size (*S*) and the rotation angle (*A*) of the hexagonal lattice cells in the studied 3D printed hexagonal prism lattice polyamide structures is schematically depicted in Fig. 2. Figures 3, 4 and 5 show examples of the manufactured 3D-printed hexagonal prism lattice polyamide structures, including the effects of cell size (Fig. 3), rotation angle of the hexagonal lattice cells (Fig. 4), and sample height (Fig. 5).

## Sound absorption coefficient measurements

**Fig. 6:**
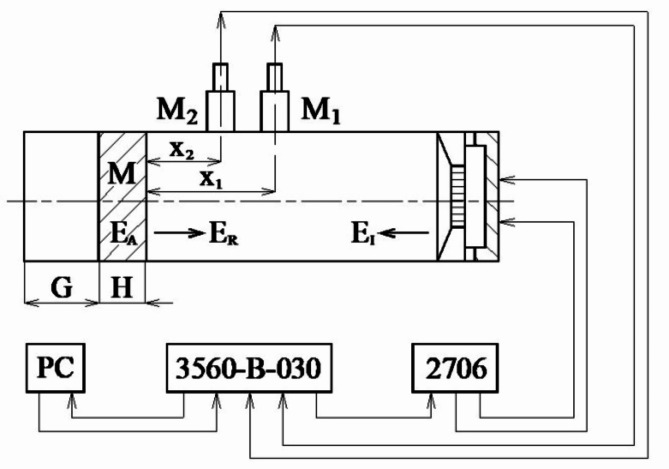
Schematic diagram of the measurement apparatus. Legend of the abbreviations: *E*_*A*_—absorbed acoustic energy; *E*_*I*_—incident acoustic energy; *E*_*R*_—reflected acoustic energy; *G*—air gap size; *M*—measured sample; *M*_1_, *M*_2_—measuring microphones; *H*—sample height (thickness); *x*_1_, *x*_2_—microphone distances from the tested sample.

The ability of materials to absorb sound is defined by the sound absorption coefficient *α*as follows^42^:

1 $$\:\alpha\:=\frac{{E}_{A}}{{E}_{I}}=1-\frac{{E}_{R}}{{E}_{I}}$$

where *E*_*A*_ is the absorbed acoustic energy, *E*_*I*_ is the incident acoustic energy, and *E*_*R*_ is the reflected acoustic energy. The sound absorption properties of the 3D printed material samples studied were measured using an acoustic impedance tube (BK 4206) in combination with a signal PULSE multi-analyser (BK 3560-B-030) and a power amplifier (BK 2706) in the frequency range *f* = (250, 6400) Hz (Brüel & Kjær, Nærum, Denmark). A schematic diagram of the measurement apparatus for measuring frequency dependencies of the sound absorption coefficient is shown in Fig. 6. The normal incidence sound absorption coefficient of the tested samples of a given height *H* was experimentally obtained for different sizes of the air gap *G* (ranging from 0 mm to 80 mm) behind the investigated specimen M, as shown in Fig. 6. The experimental measurements were carried out at an ambient temperature of 20 °C.

Frequency dependencies of the normal incidence sound absorption coefficient of the investigated 3D printed specimens were determined based on the partial standing wave principle using the transfer function method^43^, which is standardized according to ISO 10534-2. In this case, the normal incidence sound absorption coefficient *α* is expressed as follows^44,45^:

2 $$\:\alpha\:=1-{\left|r\right|}^{2}$$

where *r* is the normal incidence reflection factor defined by the equation:

3 $$\:r={r}_{r}+i{r}_{i}=\frac{{H}_{12}-{H}_{I}}{{H}_{R}-{H}_{12}}\cdot\:{e}^{2k\cdot\:{x}_{1}i}$$

where *r*_*r*_ and *r*_*i*_ are the real and imaginary components of the normal incidence reflection factor *r*, *H*_12_ is the complex acoustic transfer function, *H*_*I*_ is the transfer function for the incident acoustic wave, *H*_*R*_ is the transfer function for the reflection acoustic wave, *k* is the wave number, and *x*_1_ is the distance between the tested material specimen and the microphone M_1_ (see Fig. 6). The transfer functions are expressed as follows:

4 $$\:{H}_{12}=\frac{{p}_{2}}{{p}_{1}}=\frac{{e}^{k\cdot\:{x}_{2}i}+r\cdot\:{e}^{-k\cdot\:{x}_{2}i}}{{e}^{k\cdot\:{x}_{1}i}+r\cdot\:{e}^{-k\cdot\:{x}_{1}i}}$$

5 $$\:{H}_{I}={e}^{-k\cdot\:\left({{x}_{1}-x}_{2}\right)i}$$

6 $$\:{H}_{R}={e}^{k\cdot\:\left({{x}_{1}-x}_{2}\right)i}$$

where *p*_1_ and *p*_2_ are the complex acoustic pressures at the two microphone positions, and *x*_2_ is the distance between the investigated material specimen and the microphone M_2_ (see Fig. 6).

As mentioned above, the sound absorption properties of materials are influenced by many factors, including the excitation frequency of acoustic waves. The effect of the excitation frequency on the sound absorption coefficient is expressed by the noise reduction coefficient *NRC*. It represents a single number ranging from 0 to 1 that describes the average sound absorption properties of a given material. It is defined as the arithmetical average of the measured sound absorption coefficients *α* at excitation frequencies of 250 Hz, 500 Hz, 1000 Hz, and 2000 Hz^46,47^:

7 $$\:NRC=\frac{{\alpha\:}_{250}+{\alpha\:}_{500}+{\alpha\:}_{1000}+{\alpha\:}_{2000}}{4}$$

The sound absorption properties of the investigated lattice samples were also compared using the mean sound absorption coefficient *α*_*m*_, which was calculated as the arithmetic mean of the sound absorption coefficients across the entire frequency range, i.e., from 250 Hz to 6400 Hz.

## Numerical simulations of specific airflow resistance

The ability of open-porous materials to damp the sound is closely related to their airflow resistivity. The important quantity to describe the ability of materials to resist airflow is the specific airflow resistance *R*_*s*_, which is defined by the formula^48,49^:

8 $$R_S=\Delta p/v=\left( \Delta p \cdot A \right)/q_v$$

where Δ*p* is the pressure difference across the test specimen, *v* is the linear airflow velocity, *A* is the specimen’s cross-sectional area, and *q*_*v*_ is the volumetric airflow rate. As is well known, an increase in airflow resistance improves sound absorption properties over the entire frequency range, but only up to an intermediate value. A too resistive porous material will exhibit poor sound absorption behaviour because it will be more difficult for the acoustic wave to penetrate through the material^50^.

Numerical simulations of the specific airflow resistance of the investigated specimens were performed using Ansys software. In these simulations, the studied 3D printed material structures were placed in the middle of a pipe with an internal diameter *d* = 28.9 mm. Additionally, the pipe was designed to be long enough depending on the specimen thickness and the volumetric airflow was stable in the tube. The computational mesh was created using Ansys Fluent Meshing software. First, a surface mesh was created from triangular elements, from which a volume mesh was generated by combining hexahedral and polyhedral elements. Subsequently, a volumetric mesh was created from the surface mesh. Furthermore, four (or eight) prismatic layers were created on the pipe wall (or on the sample structure). It was also necessary to determine the type of airflow inside the pipe using the Reynolds number *Re*^51^:

9 $$\:Re=\frac{v\cdot\:d}{\nu}$$

where *ν* is the kinematic viscosity of air at a temperature of 20 °C (i.e., *ν* = 1.51⋅10^−5^ m^2^⋅s^−1^). According to EN 29053^52^, the simulations of the specific airflow resistance of samples with an outside diameter of 28.9 mm (as in the case of the sound absorption measurements) were carried out in the range of velocities *v* = (0.001, 0.050) m⋅s^−1^. The maximum Reynolds number *Re*_*max*_ = 95.6 was obtained for the maximum airflow velocity (i.e., *v*_*max*_ = 0.05 m⋅s^−1^) and is significantly lower compared to the critical Reynolds number *Re*_*crit *_(i.e., 2320 in pipe flow). Therefore, it is assumed that the airflow in the pipe is laminar^53^, and a laminar viscous model was used in these simulations. Due to the small flow velocities and small pressure differences, the air compressibility was neglected. The “Velocity inlet” and “Pressure output” boundary conditions were set for the pipe´s inlet and outlet, with a static pressure value of 0 Pa. The pipe walls were set as stationary with a “No slip” condition. “Symmetry” boundary conditions were imposed on the symmetry surfaces.

## Results and discussion

### Frequency dependencies of the sound absorption coefficient

This section deals with the factors affecting the sound absorption properties of the studied 3D-printed hexagonal prism lattice polyamide materials. Specifically, it focuses on the influence of sample height (*H*), rotation angle (*A*) of the hexagonal lattice cells, cell size (*S*), air gap size (*G*) behind the acoustic impedance tube (see Fig. 6), outer shell thickness (*W*), and the excitation frequency (*f*) of acoustic waves.

**Fig. 7:**
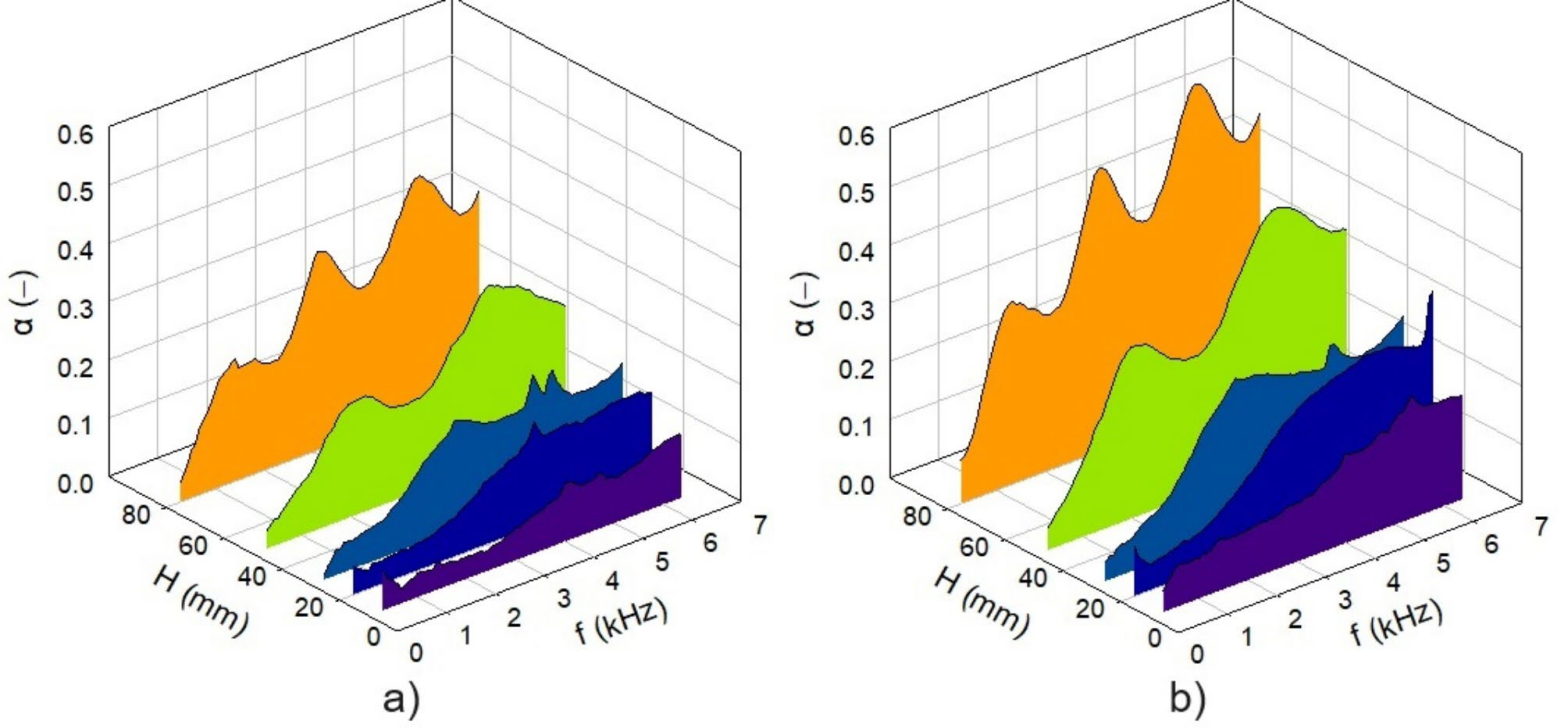
Effect of the sample height *H* on the frequency dependencies of the sound absorption coefficient for the investigated polyamide samples: (**a**) Hx_A0_S5_G0 and (**b**) Hx_A30_S10_G0.

The effect of varying the sample height *H* (or the thickness) on sound absorption properties of the studied 3D-printed hexagonal prism lattice polyamide material structures is shown in Fig. 7. It is evident that the material’s ability to absorb sound generally increased with the sample height, regardless of the other sample parameters (i.e., *A*, *S*, and *G*), as shown in Fig. 7a and b. Therefore, the samples with a maximum height of 80 mm exhibited the best sound absorption properties. However, increasing the height of the samples leads to higher production costs and longer time requirements^54^ for the 3D-printed polyamide material structures.

**Fig. 8:**
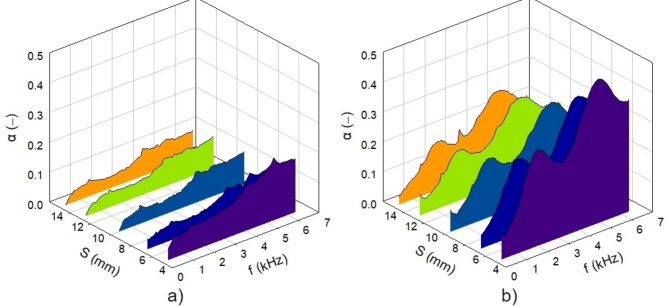
Effect of the cell size *S* on the frequency dependencies of the sound absorption coefficient for the investigated polyamide samples: (**a**) H10_A0_Sx_G0 and (**b**) H50_A45_Sx_G0.

The cell size *S *of the investigated 3D-printed open-porous lattice polyamide samples belongs to the significant factors affecting their sound absorption performance. Generally, a decrease in cell size results in higher density (or lower total volume porosity) of open porous materials. This is accompanied by higher air-flow resistivity, leading to improved sound absorption properties of higher-density open-porous materials^55^. This phenomenon is evident in Fig. 8, where the sound absorption properties generally improved as the cell size of the tested lattice polyamide samples decreased, regardless of other sample parameters (i.e., *H*, *A*, and *G*). For this reason, the samples produced with the smallest cell size (i.e., 5 mm) exhibited the best sound absorption performance.

**Fig. 9:**
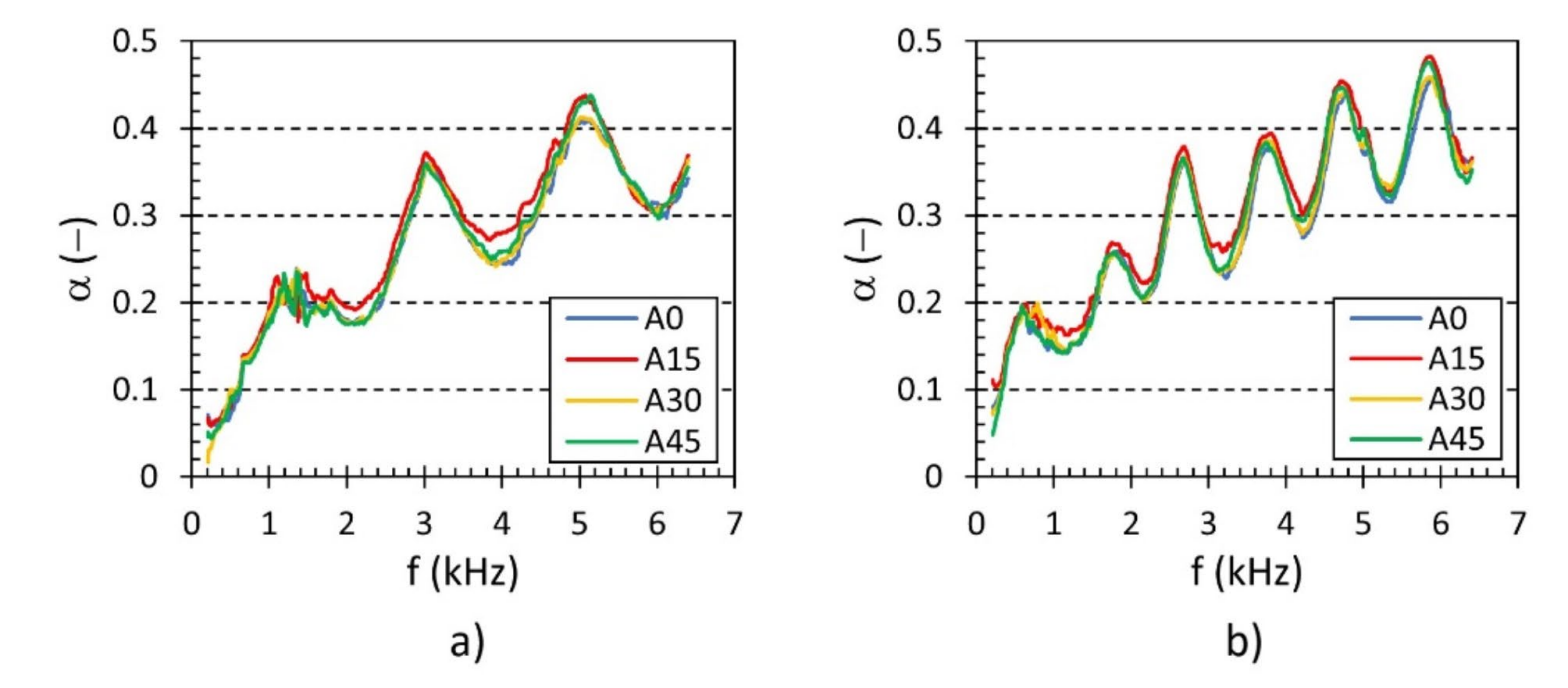
Effect of the rotation angle *A* of hexagonal lattice cells on the frequency dependencies of the sound absorption coefficient for the investigated polyamide samples: (**a**) H80_Ax_S10_G0 and (**b**) H80_Ax_S10_G80.

The effect of the rotation angle *A* of the hexagonal lattice cells on the sound absorption performance for two different open-porous lattice polyamide samples is shown in Fig. 9a and b. It is visible that the samples produced with the rotation angle of 15° of hexagonal lattice cells exhibited slightly better sound absorption properties compared to those produced with other cell angles. However, the effect of the rotation angle is practically negligible in the whole measured frequency range. Similar results were also obtained for the other samples investigated.

**Fig. 10:**
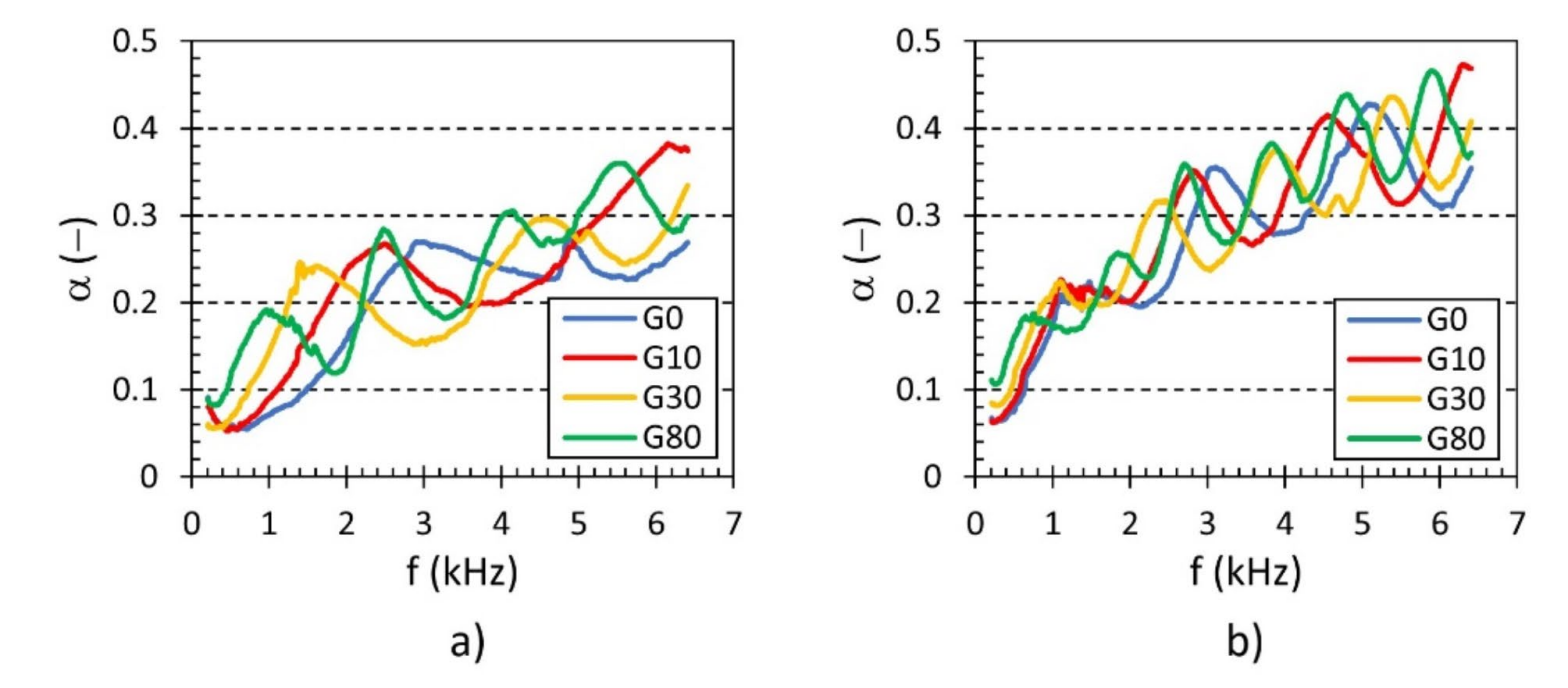
Effect of the air gap size *G* on the frequency dependencies of the sound absorption coefficient for the investigated polyamide samples: (**a**) H30_A15_S5_Gx and (**b**) H80_A15_S10_Gx.

As shown in Fig. 10, the air gap size *G* behind the investigated samples (see Fig. 6) had a significant influence on their sound absorption performance. Over the entire frequency range, a certain number of minima and maxima of the sound absorption coefficient can be observed. This phenomenon can be attributed to sound reflections from the solid wall inside the impedance tube behind the tested specimen, and to the wavelength *λ*, which is given by the ratio of the speed of sound to the frequency ^56^. At the wall surface, the sound pressure reaches a maximum value while the air particle velocity is zero. Conversely, at a quarter wavelength distance from a solid wall, the sound pressure is zero and the air particle velocity reaches a maximum. Placing the investigated porous material at a quarter wavelength (i.e., *λ*/4) away from the solid wall allows for maximum sound absorption due to the maximum air particle velocity. Similarly, at a half-wavelength (i.e., *λ*/2), both the air particle velocity and the sound absorption coefficient reach their minimum values. It is clear from the above that maximum values of the sound absorption coefficient occur at odd multiples of quarter wavelengths in the standing wave antinodes at the frequencies:

10 $$\:f=\frac{c\cdot\:\left(2n+1\right)}{4\cdot\:\left(G+H/2\right)}$$

where *c* is the speed of sound, and *n* is an integer (*n* = 0, 1, 2…). Similarly, minimum values of the sound absorption coefficient occur at even multiples of quarter wavelengths in standing-wave nodes at the frequencies:

11 $$\:f=\frac{c\cdot\:n}{2\cdot\:\left(G+H/2\right)}$$

Figure 10a and b illustrate that the number of sound absorption maxima (*α*_*max*_) and minima (*α*_*min*_) at the corresponding frequencies (*f*_*max*_ and *f*_*min*_) generally increased with increasing the air gap size *G* behind the tested specimens inside the impedance tube. Therefore, increasing the air gap size enhances the sound absorption properties of open-porous materials, especially at low frequencies. This method of increasing the sound absorption at low sound frequencies is more effective than increasing the sample material’s height, resulting in shorter printing times and lower production costs for 3D-printed samples.

**Fig. 11:**
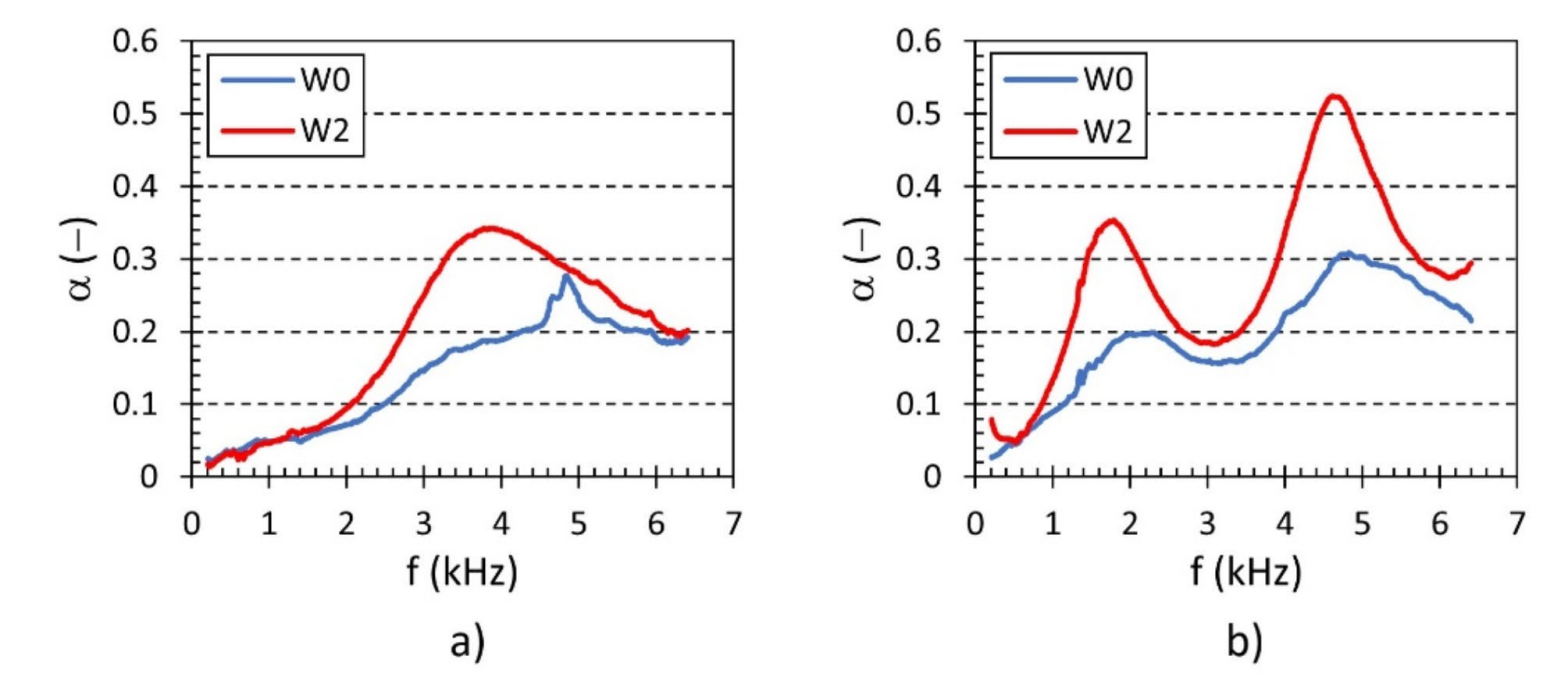
Effect of the outer shell on the frequency dependencies of the sound absorption coefficient for the investigated polyamide samples: (**a**) H20_A45_S7_G0 and (**b**) H50_A30_S10_G0.

As mentioned above, the studied samples were produced both with and without an outer shell, which had a wall thickness *W* = 2 mm, as shown in Fig. 1. Examples of the effect of the outer shell on the frequency dependencies of the sound absorption coefficient are shown in Fig. 11. It is obvious that the lattice samples with the full outer shell (i.e., W2) exhibited better sound absorption properties compared to those without the outer shell (i.e., W0). This is due to a higher airflow resistance, where higher air velocity in smaller cross-sections (i.e., W2 samples) at a given airflow rate causes higher pressure drops as air flows through the material structure, leading to a higher conversion of air pressure energy into heat. Similarly, there is a higher conversion of acoustic energy into heat when acoustic waves propagate through samples made with the outer shell.

The data from Figs. 7, 8, 9, 10 and 11 clearly demonstrate that the excitation frequency *f* of acoustic waves significantly impacts the sound absorption behavior of the studied 3D-printed hexagonal prism lattice polyamide material structures. It was found that higher excitation frequencies led to better sound absorption properties. To enhance sound absorption at lower frequencies, increasing the sample height *H* or the air gap size *G* behind the specimen inside the acoustic impedance tube can be an effective way to increase the sound absorption.

## Mathematical simulations of specific airflow resistance

This chapter deals with different factors that influence the specific airflow resistance (*R*_*s*_) of the studied 3D-printed hexagonal prism lattice polyamide material structures, which has been numerically simulated using Ansys software. In addition, the specific airflow resistances were compared with the noise reduction coefficient, which was determined based on Eq. (7) from the measured frequency dependencies of the sound absorption coefficient and for the maximum air gap size (i.e., *G* = 80 mm) behind the tested samples inside the acoustic impedance tube.

**Fig. 12:**
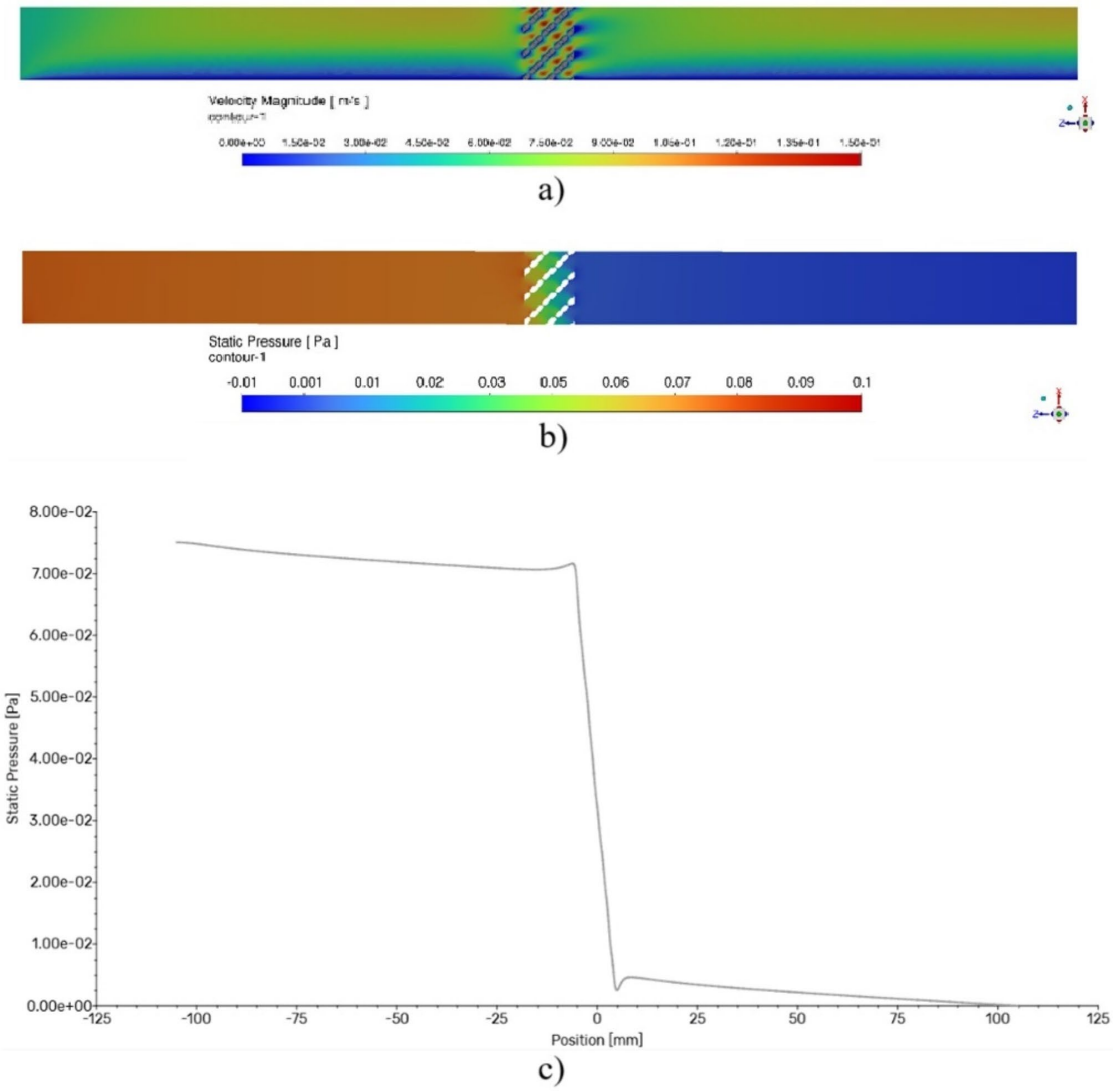
Numerical simulation of air flow through the porous sample H10_A0_S5: (**a**) velocity magnitude profile, (**b**) static pressure profile and (**c**) static pressure vs. pipe position dependence.

The principle of mathematical simulation of the specific airflow resistance using Ansys software is demonstrated in Fig. 12. Figure 12a and b present the velocity magnitude profile and static pressure profile during airflow with a mean velocity of 0.05 m⋅s^−1^ through the investigated 3D-printed porous sample, specifically type H10_A0_S5, within the pipe. Figure 12c shows the variation in static pressure along the pipe. It is evident that the air static pressure decreased significantly as the airflow passes through the porous sample due to the sample’s airflow resistivity. Consequently, airflow at a given velocity *v* through the sample is accompanied by a corresponding pressure difference (i.e., pressure drop) Δ*p*.

As mentioned above, the numerical simulations were conducted across a range of airflow velocities, specifically *v* = (0.001, 0.050) m⋅s^−1^, to determine the corresponding pressure differences Δ*p* through the porous samples under investigation. Subsequently, based on these simulations, the specific airflow resistance of the studied 3D-printed hexagonal prism lattice material structures was determined. As demonstrated by Eq. (8) above, the specific airflow resistance is proportional to the ratio of the pressure difference Δ*p* to the linear airflow velocity *v* during airflow through the test specimen. Therefore, the specific airflow resistance is given by the slope of the line from a linearized Δ*p*-*v* dependence.

**Fig. 13:**
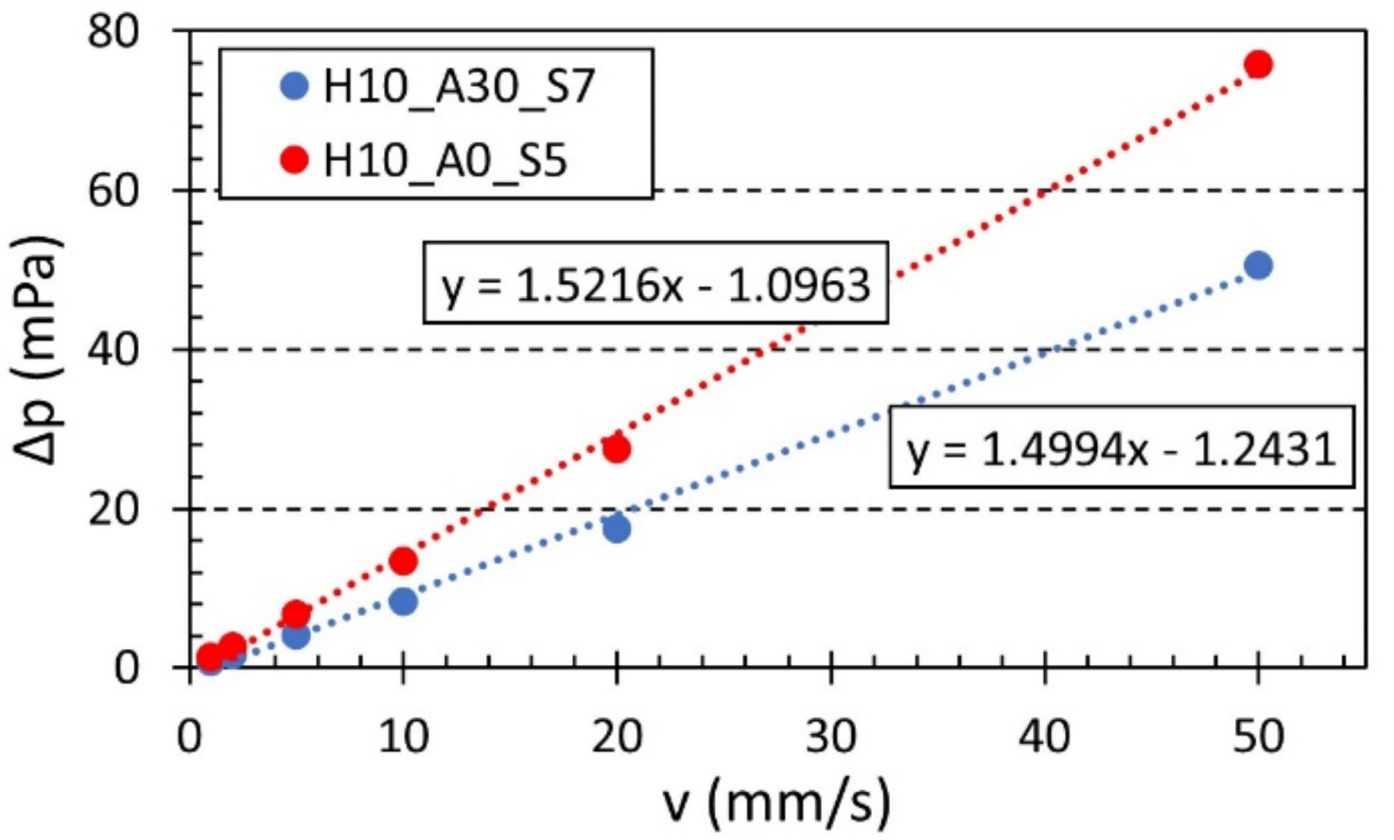
Numerically simulated pressure difference vs. airflow velocity dependencies for the investigated polyamide samples H10_A0_S5 and H10_A30_S7.

Figure 13 shows examples of numerically simulated specific airflow resistances. It can be seen that the sample type H10_A0_S5 exhibited a higher specific airflow resistance (i.e., 1.5216 Pa∙s/m) compared to the sample type H10_A30_S7 (i.e., 1.4994 Pa∙s/m). Therefore, the sample H10_A0_S5 has a higher acoustic resistance (and better sound absorption properties) to acoustic wave propagation through its material structure compared to the sample H10_A30_S7.

**Fig. 14:**
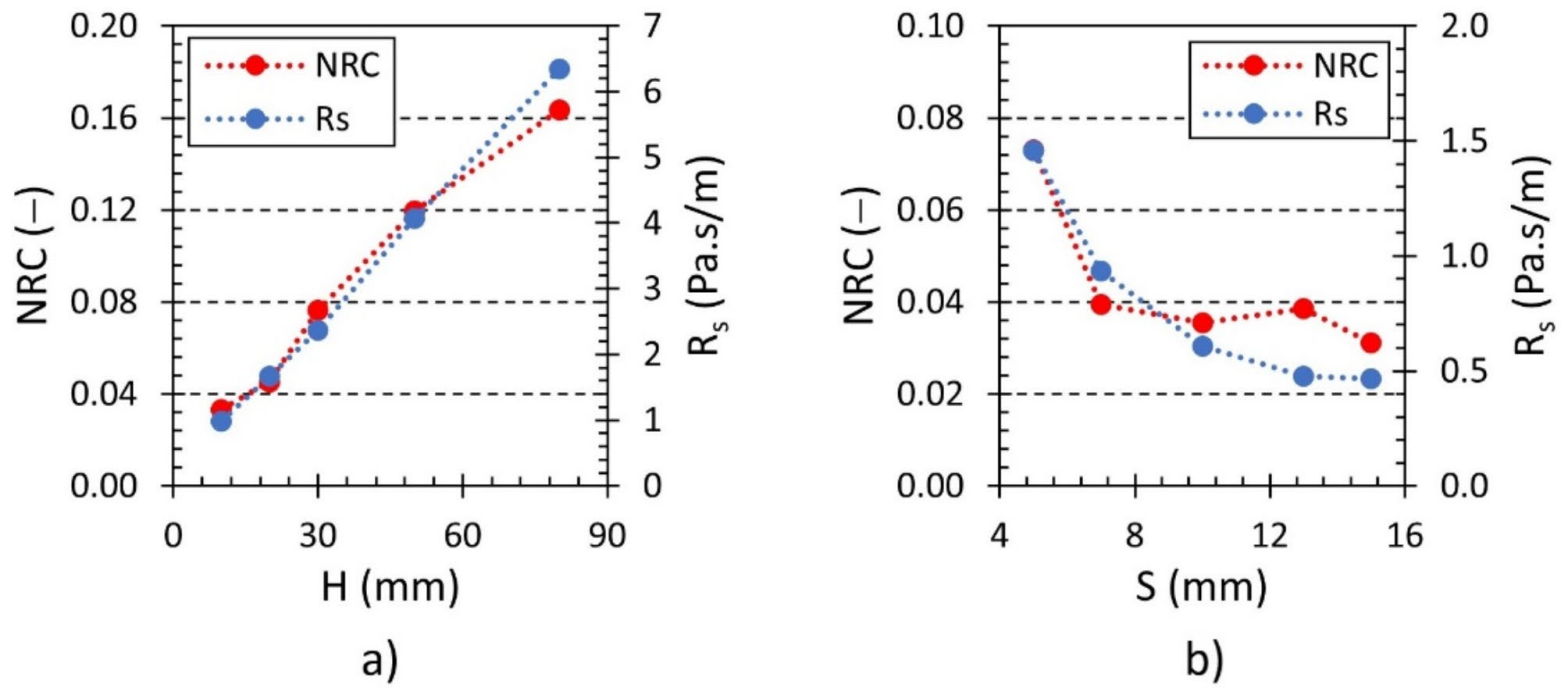
Effect of the sample height *H* and cell size *S* on the specific airflow resistance *R*_*s*_ and the noise reduction coefficient *NRC* for the investigated polyamide samples: (**a**) Hx_A30_S7 and (**b**) H10_A0_Sx.

The influence of sample height and the cell size on the specific airflow resistance *R*_*s*_ and noise reduction coefficient (*NRC*) is depicted in Fig. 14. It is evident that both *R*_*s*_ and *NRC* increase with increasing sample height *H* (see Fig. 14a). Therefore, a higher specimen height led to increased airflow resistance and, consequently, enhanced sound absorption properties of the studied 3D-printed hexagonal prism lattice polyamide material structures. Contrarily, as shown in Fig. 14b, the increasing cell size *S* (or the increasing sample´s volume porosity) generally led to decrease in the airflow resistance *R*_*s*_ and the coefficient *NRC*.

**Fig. 15:**
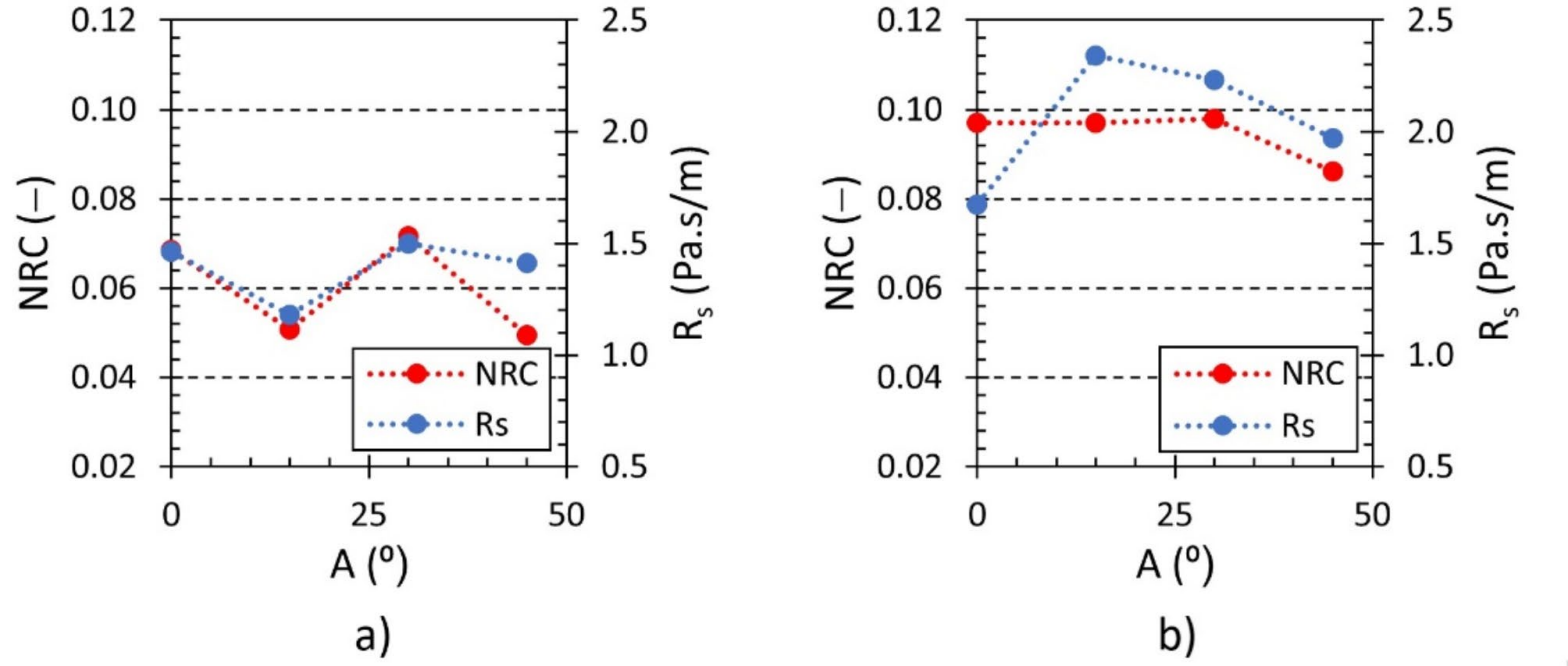
Effect of the rotation angle *A* of hexagonal lattice cells and the outer sample´s shell on the specific airflow resistance *R*_*s*_ and the noise reduction coefficient *NRC* for the investigated polyamide samples H10_Ax_S10: (**a**) without outer shell and (**b**) with outer shell.

The effect of the rotation angle *A* of hexagonal lattice cells and the outer shell on the specific airflow resistance *R*_*s*_ and the noise reduction coefficient *NRC* is shown in Fig. 15. It is obvious that the effect of the rotation angle on the airflow resistance *R*_*s*_ and the coefficient *NRC* was not clear as well as in the above-mentioned frequency dependencies of the sound absorption coefficient. However, it can be observed that a higher value of the airflow resistance *R*_*s*_ generally resulted in a higher *NRC* coefficient and, therefore, to better sound absorption properties (see Fig. 15a). It is also evident that higher values of *R*_*s*_ and *NRC* were observed in samples fitted with the outer shell (see Fig. 15b) compared to those without the outer shell, as shown in Fig. 15a.

It can be concluded from the above that a higher value of the specific airflow resistance *R*_*s*_ of the studied 3D-printed hexagonal prism lattice polyamide material specimens generally led to a higher value of the noise reduction coefficient *NRC*, and thus, to better sound absorption properties of the investigated specimens. For these reasons, the mathematically simulated specific airflow resistances are in an excellent agreement with the experimentally measured frequency dependencies of the sound absorption coefficient.

## Summary

As previously mentioned, the noise reduction coefficient (*NRC*) quantifies the average sound absorption properties of the material specimens under investigation across a frequency range of 250 Hz to 2000 Hz. Maximum values of this coefficient (i.e., *NRC*_*max*_), considering variables such as sample height (*H*), air gap size (*G*), and the presence of the outer shell, were calculated using Eq. (7) with appropriate cell sizes (*S*) and rotation angles (*A*) of the hexagonal lattice cells. These values are given in Table 3. Notably, the highest *NRC* value, (i.e., *NRC*_*max*_ = 0.5118), was achieved by the sample type H80_A30_S5, which included the outer shell and had an air gap size of *G* = 10 mm. It was observed that the *NRC* coefficient tends to increase with higher sample heights, air gap sizes, and smaller cell sizes, especially for samples equipped with an outer shell of thickness *W* = 2 mm. However, the effect of the angle of rotation (*A*) of the hexagonal lattice cells on the *NRC* coefficient was not entirely clear.

**Table 3:** Maximum values of the noise reduction coefficient.

*H* (mm)	*W* (mm)		*G* (mm)			
			**0**	**10**	**30**	**80**
10	0	Sample	H10_A0_S5	H10_A0_S5	H10_A0_S5	H10_A0_S5
		*NRC*_*max*_	0.073	0.0789	0.0974	0.0916
	2	Sample	H10_A15_S5	H10_A45_S5	H10_A30_S5	H10_A45_S5
		*NRC*_*max*_	0.0532	0.0665	0.1117	0.1052
20	0	Sample	H20_A45_S5	H20_A45_S5	H20_A30_S5	H20_A0_S5
		*NRC*_*max*_	0.0561	0.0782	0.1209	0.1142
	2	Sample	H20_A0_S5	H20_A0_S5	H20_A0_S5	H20_A0_S5
		*NRC*_*max*_	0.0892	0.1835	0.1801	0.1756
30	0	Sample	H30_A45_S5	H30_A45_S5	H30_A45_S5	H30_A45_S5
		*NRC*_*max*_	0.1084	0.1308	0.1498	0.1822
	2	Sample	H30_A15_S5	H30_A45_S5	H30_A30_S5	H30_A0_S5
		*NRC*_*max*_	0.1870	0.1827	0.2280	0.2014
50	0	Sample	H50_A0_S5	H50_A30_S5	H50_A30_S5	H50_A45_S5
		*NRC*_*max*_	0.16	0.1665	0.1815	0.2298
	2	Sample	H50_A15_S5	H50_A15_S5	H50_A15_S5	H50_A45_S5
		*NRC*_*max*_	0.2687	0.2805	0.2641	0.3966
80	0	Sample	H80_A30_S5	H80_A30_S5	H80_A30_S5	H80_A0_S5
		*NRC*_*max*_	0.2061	0.2202	0.2795	0.2477
	2	Sample	H80_A30_S5	H80_A30_S5	H80_A30_S5	H80_A30_S5
		*NRC*_*max*_	0.4802	0.5118	0.4587	0.4702

The sound absorption properties of the investigated lattice samples were also compared using the mean sound absorption coefficient *α*_*m*_ over the entire measured frequency range (i.e., from 250 Hz to 6400 Hz). The sample types that exhibited the maximum value of the coefficient *α*_*m*_ under specific conditions (such as height *H*, wall thickness *W*, and air gap sizes of 0 mm and 80 mm) are given in Tables 4 and 5. These tables also include the *NRC* coefficient, and the maximum measured value of the sound absorption coefficient (*α*_*max*_) at the corresponding frequency (*f*_*max*_). Once again, it is clear that the sound absorption properties tend to improve with increased sample heights, higher excitation frequencies, and smaller cell sizes, especially for samples equipped with an outer shell thickness of *W* = 2 mm. The impact of the rotation angle of the hexagonal lattice cells on these properties remains ambiguous. The highest value of the mean sound absorption coefficient (i.e., *α*_*m*_ ≅ 0.775) and therefore the best sound absorption properties were found for the sample H80_A30_S5, which was fitted with a 2 mm thick outer shell, as shown in Tables 4 and 5. The noise reduction coefficient *NRC* of this sample exhibited the highest value among all the measured samples, ranging from 0.46 to 0.51, regardless of the air gap size, as presented in Table 3.

From the above, it can be concluded that the experimentally measured dependencies of sound absorption and the derived parameters (specifically, the coefficients *NRC* and *α*_*m*_) were in excellent agreement with the numerically simulated specific airflow resistances *R*_*s*_.

**Table 4:** Maximum values of the mean sound absorption coefficient and further parameters for the air gap size *G* = 0 mm.

H (mm)	W (mm)	Sample	α_mmax_ (−)	NRC (−)	α_max_ (−)	f_αmax_ (Hz)
10	0	H10_A0_S5	0.1267	0.0730	0.2470	5248
	2	H10_A45_S5	0.1468	0.0450	0.3890	6288
20	0	H20_A30_S5	0.1828	0.0510	0.4966	5744
	2	H20_A0_S5	0.3542	0.0892	0.6734	3136
30	0	H30_A45_S5	0.2691	0.1084	0.5254	4944
	2	H30_A45_S5	0.4121	0.1512	0.8064	6288
50	0	H50_A45_S5	0.3181	0.1543	0.5002	4712
	2	H50_A15_S5	0.5584	0.2687	0.9428	6400
80	0	H80_A30_S5	0.4052	0.2061	0.6336	4832
	2	H80_A30_S5	0.7793	0.4802	0.9928	5264

**Table 5:** Maximum values of the mean sound absorption coefficient and further parameters for the air gap size *G* = 80 mm.

H (mm)	W (mm)	Sample	α_mmax_ (−)	NRC (−)	α_max_ (−)	f_αmax_ (Hz)
10	0	H10_A0_S5	0.1927	0.0916	0.4269	5280
	2	H10_A0_S5	0.1812	0.0951	0.3694	4560
20	0	H20_A30_S5	0.2034	0.0976	0.3897	4856
	2	H20_A0_S5	0.3027	0.1756	0.8059	5984
30	0	H30_A45_S5	0.3006	0.1822	0.5018	5344
	2	H30_A45_S5	0.4045	0.1913	0.8144	6392
50	0	H50_A45_S5	0.3486	0.2298	0.5583	5808
	2	H50_A15_S5	0.5847	0.3900	0.9955	4968
80	0	H80_A30_S5	0.4284	0.2412	0.6637	5680
	2	H80_A30_S5	0.7721	0.4702	0.9998	2944

## Conclusions

The investigation presented in this paper is focused on the sound absorption properties of 3D-printed open-porous polyamide hexagonal prism lattice structures, which were produced with varying sizes and rotation angles of the lattice cells, as well as with different heights. These specimens were also fabricated with an outer shell. The evaluation of their sound absorption properties was conducted by measuring the normal incidence sound absorption coefficient in an acoustic impedance tube. In addition, the impact of the air gap size behind the samples within the impedance tube on their sound absorption was investigated. The ability of the samples to absorb sound was further compared to their specific airflow resistance, which was numerically simulated using Ansys software.

This study found that the above factors significantly influenced the sound absorption properties and specific air flow resistance of the open-porous lattice structures under investigation. It can be concluded that the material´s ability to absorb sound (indicated by the noise reduction coefficient *NRC* and mean sound absorption coefficient *α*_*m*_) tends to increase with increasing sample height, excitation frequency, and air gap size behind samples tested inside the acoustic impedance tube, as well as with decreasing lattice cells’ size. In addition, specimens equipped with an outer shell 2 mm thick demonstrated superior sound absorption properties compared to those fully structured as a lattice. The rotation angle of the lattice cells was found to have a negligible impact on sound absorption. The optimal sound absorption characterized by *NRC* values ranging from 0.46 to 0.51 and a mean sound absorption coefficient of 0.775, was observed for the specimen measuring 80 mm in height, with a cell size of 5 mm, a lattice cell rotation angle of 30°, and an outer shell thickness of 2 mm, regardless of the air gap size.

Similar results were also confirmed by numerical simulations of the specific air flow resistance, which generally increased with improved sound absorption properties of the investigated samples. It can be stated that the numerically simulated specific air flow resistances were in excellent agreement with the experimentally measured sound absorption properties of the open-porous hexagonal prism lattice structures. Consequently, numerical simulations of the specific airflow resistivity of 3D-printed open-porous material structures can be beneficial in developing innovative 3D-printed materials for sound absorption before their production.

The application of open-porous, lightweight 3D-printed materials shows great promise in reducing material weight, saving time, and conserving energy. Other advantages of these materials include their resistance to moisture and chemicals, variable density, and recyclability, making 3D-printed materials competitive with commonly used sound-absorbing materials such as polyurethane foam, glass wool, and mineral wool. In the future, it will be possible to develop advanced lightweight 3D-printed structures that are unattainable through conventional manufacturing methods. The results also highlight the potential of 3D-printed structures for noise reduction, due to their rapid production and high degree of customization, allowing the creation of complex geometries that can be seamlessly integrated into existing systems or infrastructures. This enables quick experimentation and verification of optimal designs. Potential applications can be dedicated to reducing unwanted noise in classrooms, conference halls, machine systems, hospitals, transportation vehicle cabins, ventilation systems, sports halls, gymnasiums, swimming pools, wellness centers, consumer electronics, and even for aesthetic purposes.

This paper presents the first scientific insights into the sound absorption properties of 3D-printed open-porous hexagonal prismatic lattice structures, which, to the best of our knowledge, have not been previously published in this context. These findings will be used to develop new, more efficient types of sound-absorbing 3D-printed open-porous hexagonal prismatic lattice structures as follows:

- Optimization of different parameters of lattice material specimens to improve sound absorption the studied hexagonal prismatic lattice structures.
- Development of a new numerical model for simulating sound absorption properties based on different material and acoustic parameters, and its comparison with experimentally measured results.
- Optimization of 3D printing conditions (e.g., layer thickness and printing temperature) to enhance the sound absorption properties of lattice material structures.
- Development of new, variable-density 3D-printed hexagonal prismatic lattice structures to control sound absorption and resonance across different frequency ranges.
- Research into new multilayer open-porous hexagonal prismatic lattice structures manufactured using 3D printing technology to enhance sound absorption properties while reducing weight and production costs.

## Data Availability

The datasets used during the current study available from the corresponding author on reasonable request.
